# Draft genome sequences of *Citrobacter cronae* isolates recovered from food animal production chain in Nigeria

**DOI:** 10.1128/mra.00201-25

**Published:** 2025-06-27

**Authors:** Ibrahim A. Raufu, Opeyemi U. Lawal, Valeria R. Parreira, Mitra Soni, Harmanpreet Kaur, Akeem O. Ahmed, Abdulfatai Aremu, Ahmad I. Al-Mustapha, Lawrence Goodridge

**Affiliations:** 1 Department of Veterinary Microbiology, University of Ilorin 108285, Ilorin, Nigeria; 2 Canadian Research Institute for Food Safety, University of Guelph 3653, Guelph, Ontario, Canada; 3 The Global Bacterial Antimicrobial Resistance Sequencing (GLOBAR-SEQ) Program, Ontario, Canada; 4 School of the Environment, University of Windsor 8637, Windsor, Ontario, Canada; 5 Department of Veterinary Pharmacology and Toxicology, University of Ilorin 108285, Ilorin, Nigeria; 6 Department of Food Hygiene and Environmental Health, Faculty of Veterinary Medicine, University of Helsinki 3835, Helsinki, Finland; 7 Department of Veterinary Services, Kwara State Ministry of Agriculture and Rural Development, Ilorin, Nigeria; Loyola University Chicago, Chicago, Illinois, USA

**Keywords:** antimicrobial resistance, zoonotic pathogens, food safety, *Citrobacter*

## Abstract

*Citrobacter cronae* inhabits the intestines of humans and has been isolated from stool and rectal swabs of immunosuppressed patients. However, its role as a zoonotic pathogen has not been fully studied. Here, we present draft genomes of 11 *C*. *cronae* isolates obtained from the food-animal production chain.

## ANNOUNCEMENT

*Citrobacter cronae* colonizes the human gut and has been isolated from stool and rectal swabs of immunocompromised patients (1). It is an emerging opportunistic pathogen linked to bacteremia, urinary tract infections, especially in hospitalized individuals (2–4). *C. cronae* is concerning due to its multidrug resistance and potential to harbor resistance genes (2, 5). Its recent detection in edible snails in Nigeria suggests animal reservoirs and zoonotic transmission routes (6). Here, we report draft genomes of *C. cronae* isolates from the food-animal production, under the Global Bacterial Antibiotic Resistance Sequencing (GLOBAR-SEQ) program, which supports genomic surveillance of pathogens in low- and middle-income countries (7, 8).

Intestinal samples from slaughterhouse processing and 200 mL water samples from poultry and cattle farms in Ilorin, Nigeria, were obtained as part of routine surveillance exempt from ethical approval. For each sample, 10 g or 10 mL was enriched in 90 mL of buffered peptone water (Fluka Biochemika, Germany) and incubated at 35°C for 20 h. One milliliter was transferred to 9 mL of Selenite-F broth (Fluka Biochemika, Germany) and incubated at 35°C for 24 h. Cultures were streaked onto *Salmonella-Shigella* agar (Laboratorios Britania, Argentina) and incubated for 20 h at 35°C. Black colonies were sub-cultured onto XLD agar (Oxoid, UK) and incubated for 20 h at 35°C. Presumptive *Salmonella/Citrobacter* isolates were sent to the Canadian Research Institute for Food Safety for sequencing. Isolates were re-cultured in tryptic soy broth and incubated at 35°C for 20 h before being streaked onto XLT-4 Agar (ThermoFisher Scientific, Canada) *Salmonella-Shigella* and Brilliance *Salmonella* agar. Pure colonies were identified as belonging to the *Citrobacter* genus using the VITEK system (Biomerieux, Canada). DNA was extracted using the DNeasy Blood and Tissue Kit (Qiagen, Germany). Genomic library was prepared using the Illumina DNA Prep Tagmentation kit. Paired-end (2 × 150 bp) sequencing was conducted on the Illumina NextSeq 1000 platform.

Sequencing of 11 *Citrobacter* isolates yielded a median of 1,874,617 paired-end reads. Raw reads were quality-filtered, trimmed, and assembled as previously described (9, 10). Assembly quality was assessed using QUAST v5.2 (11). Genome annotation was performed using the NCBI Prokaryotic Genome Annotation Pipeline v6.9 (12), and resistome was determined using the CARD v4.0.0 database (13). Taxonomic classification was performed using average nucleotide identity with JSpeciesWS (14), and isolates were identified as *C. cronae*. Genome quality and completeness were assessed using CheckM2 v1.1.0 (15). All bioinformatics tools were used with default parameters.

The draft genomes of *C. cronae* yielded an average of 50 contigs, 52.18% GC content, an N50 of 213,628 bp, and a genome size of 4,872,068 bp, genome coverage ranging from 77 to 136× and 100% genome completeness. The genomes contained an average of 4,583 protein-coding sequences, 73 tRNAs, and 91 pseudogenes. Isolates carried resistance genes to nine different antibiotic classes, including aminoglycosides, quinolones, sulfonamides, macrolides, and tetracyclines. Notably, one isolate from cattle intestine carried resistance genes to eight antibiotic classes (Table 1), suggesting that *C. cronae* may act as a reservoir for antimicrobial resistance genes in the One Health continuum. These draft genomes should contribute to comparative analysis and evolutionary studies of this bacterium.

**TABLE 1:** Summary of sequence metrics of the 11 *Citrobacter cronae* isolates recovered from food animal production chain

ID	City, country	Year	Source	Specimen type	Reads	No. of contigs	Genome size (bp)	% GC	N50 (bp)	Coverage	% Genome completeness	Resistome	No. of tRNAs	Coding sequences	No. of pseudogenes	SRA	GenBank
N_SE_134	Ilorin, Nigeria	2018	Chicken slaughter slab	Intestine	2031188	58	4831711	52.22	204807	113.84	100	*aph(3')-Ia, blaCMY-159, qnrB12, qnrS1, aadA5, drfA17, floR, qacEdelta1, sul1, tet(A*)	73	4607	98	SRR32116391	JBLVHV000000000
N_SE_159	Ilorin, Nigeria	2018	Chicken slaughter slab	Liver	2255269	55	4888367	52.22	210171	124.95	100	*aph(3')-Ia, blaCMY-159, qnrB12, qnrS1, aadA5, drfA17, floR, qacEdelta1, sul1, tet(A*)	73	4652	97	SRR32116390	JBLVHU000000000
N_SE_160	Ilorin, Nigeria	2018	Chicken slaughter slab	Spleen	1395893	51	4900509	52.22	211380	77.24	100	*aph(3')-Ia, blaCMY-159, qnrB12, qnrS1, aadA5, drfA17, floR, qacEdelta1, sul1, tet(A*)	74	4666	95	SRR32116387	JBLVHT000000000
N_SE_166	Ilorin, Nigeria	2019	Chicken slaughter slab	Intestine	1786047	57	4879376	52.22	204607	100.93	100	*aph(3')-Ia, blaCMY-159, qnrB12, qnrS1, aadA5, drfA17, floR, qacEdelta1, sul1, tet(A*)	73	4653	101	SRR32116386	JBLVHS000000000
N_SE_176	Ilorin, Nigeria	2019	Chicken slaughter slab	Intestine	1874617	55	4912811	52.21	211380	104.18	100	*aph(3')-Ia, blaCMY-159, qnrB12, qnrS1, aadA5, drfA17, floR, qacEdelta1, sul1, tet(A*)	74	4680	100	SRR32116385	JBLVHR000000000
N_SE_238	Ilorin, Nigeria	2019	Chicken slaughter slab	Intestine	1753531	57	4881158	52.22	204363	99.37	100	*aph(3')-Ia, blaCMY-159, qnrB12, qnrS1, aadA5, drfA17, floR, qacEdelta1, sul1, tet(A*)	71	4649	100	SRR32116383	JBLVHP000000000
N_SE_239	Ilorin, Nigeria	2019	Chicken slaughter slab	Spleen	1667325	43	4744382	52.26	219989	95.53	100	*blaCMY-159, qnrB12*	74	4489	77	SRR32116382	JBLVHO000000000
N_SE_241	Ilorin, Nigeria	2019	Chicken slaughter slab	Intestine	1907709	52	4870308	52.23	210170	106.58	100	*aph(3')-Ia, blaCMY-159, qnrB12, qnrS1, aadA5, drfA17, floR, qacEdelta1, sul1, tet(A*)	72	4638	99	SRR32116381	JBLVHN000000000
N_SE_248	Ilorin, Nigeria	2018	Chicken slaughter slab	Intestine	1986781	44	4795929	52.26	221447	113.62	100	*aph(3')-Ia, blaCMY-159, qnrB12, qnrS1, aadA5, drfA17, floR, qacEdelta1, sul1, tet(A*)	73	4551	82	SRR32116380	JBLVHM000000000
N_SE_250	Ilorin, Nigeria	2018	Farm environment	Water	1826310	46	4795389	52.26	182686	104.75	100	*aph(3')-Ia, blaCMY-159, qnrB12, qnrS1, aadA5, drfA17, floR, qacEdelta1, sul1, tet(A*)	72	4551	82	SRR32116389	JBLVHL000000000
N_SE_264	Ilorin, Nigeria	2019	Cattle abbattoir	Intestine	3177633	40	5117803	52.02	297839	136.43	100	*aac (3)-IId, aph(3")-Ib, aph(3')-Ia, aph (6)-Id, blaCMY-159, blaTEM-1, mrx, qnrB12, qnrB5, aadA2, drfA12, mphA qacEdelta1, sul1, sul2, tet(B*)	71	4888	71	SRR32116388	JBLVHK000000000

## Data Availability

This Whole-Genome Shotgun project has been deposited at DDBJ/ENA/GenBank under the BioProject accession number PRJNA1215418. SRA and assembly accessions are listed in Table 1
